# Plant-Based Dietary Protein Is Associated with Lower Metabolic Syndrome Risk in Division III Female Athletes: A Pilot Study

**DOI:** 10.3390/nu16203486

**Published:** 2024-10-14

**Authors:** Christopher J. Kotarsky, Marissa L. Frenett, William F. Hoerle, Jiseung Kim, Jillian Lockwood, Liala Cryer, Stephen J. Ives

**Affiliations:** 1 Department of Rehabilitation, Exercise and Nutrition Sciences, University of Cincinnati, Cincinnati, OH 45267, USA; 2 Department of Health and Human Physiological Sciences, Skidmore College, Saratoga Springs, NY 12866, USA

**Keywords:** athlete, female, sports nutrition, protein pacing, metabolic syndrome

## Abstract

Background: College athletes are often overlooked for metabolic syndrome (MetS), as their increased physical activity is assumed to reduce their disease risk. However, energy or macronutrient imbalance has been shown to increase risk independent of activity. The purpose of this investigation was to assess the current dietary habits of Division III female athletes and determine their associations with body composition and MetS. Secondly, we sought to determine whether dietary intake and dietary protein source (i.e., animal- and plant-based, ABP and PBP) and quality were associated with MetS, as estimated by the Simple Method for Quantifying Metabolic Syndrome (siMS) score and the siMS risk score, and whether protein pacing was associated with body composition in Division III female athletes. Methods: Stepwise linear regression determined whether age (years), body mass (kg), body mass index (BMI; kg/m^2^), ABP (g/d), PBP (g/d), ABP:PBP, ratio of high-quality to low-quality ABP (ABP QR), relative energy intake (kcal/kg/d), and relative protein, carbohydrate, and fat intake (g/kg/d) were predictors of siMS score and siMS risk score. Results: Twenty-five athletes (19.6 ± 1.3 years; 65.9 ± 7.0 kg; 23.5 ± 2.0 kg/m^2^; ABP 71.7 ± 28.2 g/d; PBP 30.0 ± 12.2 g/d) were included in the analyses. An inverse relationship was observed between PBP and the siMS score (F_1, 22_ = 5.498, *p* = 0.028) and siMS risk score (F_1, 22_ = 7.614, *p* = 0.011). The models explained 20% and 26% of the variance in siMS score and siMS risk score, respectively. Conclusions: PBP was associated with lower MetS risk in Division III female athletes, while ABP, regardless of quality, was unrelated. These associations were independent of physical activity in this cohort of Division III female athletes.

## 1. Introduction

In the United States (US), metabolic syndrome (MetS) is a cluster of risk factors (i.e., waist circumference ≥ 35 inches or 88 cm for females, triglycerides ≥ 150 mg/dL or 1.7 mM, high-density lipoprotein < 50 mg/dL or 1.03 mM, systolic blood pressure ≥ 130 mm Hg and/or diastolic ≥ 85 mm Hg, and blood glucose > 100 mg/dL or 5.6 mM) associated with increased risk of cardiovascular disease (CVD) and all-cause mortality [1,2,3]. The prevalence of MetS increases with age and has significantly increased among US young adults, making it a major health concern for the nation [1,2,3]. Poor lifestyle choices, such as a lack of physical activity and multilayered dietary imbalances, leading to increased adipose tissue, particularly in the abdominal region, and subsequent insulin resistance, are often regarded as the main contributors to MetS and are a probable cause for the increased prevalence of MetS among younger adults [4,5,6]. This is important, as poor lifestyle habits (i.e., sedentary behavior and unhealthy dietary practices) adopted by emerging adults (i.e., 18–25 years) will likely become cemented in early adulthood, increasing the risk of developing CVD and type 2 diabetes later in life [5,7,8]. Thus, baseline MetS risk, even if low in youth, may present a trajectory in progressing through adulthood; thus, understanding MetS risk even in young, otherwise relatively healthy people, is of prognostic value [9,10,11].

College athletes are often overlooked for the diagnosis of MetS, given their focus on exercise and assumed healthier dietary habits, which are believed to reduce their disease risk [8,12]. However, overall nutritional knowledge is severely lacking in athletes [13], leading to the adoption of inappropriate and dysfunctional eating behaviors and subsequent failure to meet the dietary requirements necessary to support their physiological demands [14,15,16]. Furthermore, most college athletes do not have access to a sports dietitian, and few educational initiatives exist to increase athletes’ nutritional knowledge and improve their dietary habits and behavior [15,17,18,19]. This is extremely concerning because dietary imbalances, regardless of physical activity, such as over- or underconsumption of energy intake, including macronutrient imbalance (i.e., inappropriate distribution of dietary carbohydrates, fats, and protein), based on body mass, height, age, and sex have been shown to increase risk of MetS, as well as injury frequency and muscle wasting [20,21,22]. For example, American male football linemen have shown an increased risk for MetS due to their larger body sizes [23]. Similarly, Chinese male and female strength-related sport athletes with higher body masses have shown greater risk for all MetS risk factors compared to athletes with lower body masses [24]. These aforementioned studies highlight that factors outside of physical activity, namely diet, sex, and body mass or composition, all play a role in MetS risk, independent of physical activity. Though physical activity has the power to reduce MetS risk, when looking at a population that is already physically active, other factors may be equally important. However, there is a paucity of data amongst Division III athletes, particularly female athletes, which is important given the sex differences in MetS risk and considering lifetime risk [9,10,11].

Division III athletes appear to consume insufficient servings of fruits and vegetables on average, likely contributing to a lack of carbohydrate consumption [25]. These results were similar in both Division II female basketball athletes and collegiate female lacrosse players, demonstrating lower carbohydrate but also protein intakes compared to recommended values [26,27]. However, the general dietary status of Division III female athletes specifically is not well known. The adoption of popular dietary trends, such as a more plant-based diet, may also exacerbate existing dietary imbalances and dysfunctional eating, and the potential negative effects of these diets (e.g., reduced protein, iron, vitamin B_12_, and calcium intake) on athletes, who have higher energy and nutrient requirements, need to be evaluated further [15,28,29]. Again, this is important given that college athletes appear to lack the nutritional understanding needed to successfully implement more plant-based options into their diet that are supportive of their competitive demands, and limited research is available on the current dietary habits and energy availability of college athletes in general, especially for female athletes who are underrepresented and at risk for conditions such as premature bone mass reduction and exercise-related fractures [16,19,29,30,31,32]. For example, by protein pacing (i.e., consuming adequate dietary protein at increased frequencies throughout the day), improvements in body composition (e.g., reduced total body fat, abdominal fat, and waist circumference, and increased lean body mass) and cardiometabolic markers of health (e.g., systolic blood pressure, diastolic blood pressure, and blood lipids) have been observed [33,34,35]. In exercise-trained females, protein pacing combined with multi-component exercise, which is typical of college athletes, elicited improved muscular endurance, strength, power, and cardiovascular health [36]. Thus, a need exists to assess the current dietary habits of college athletes, specifically female athletes, and other various sex-specific factors, on sports physiology, performance, and cardiometabolic health [30,31].

The purpose of this investigation was to assess the current dietary habits of Division III female athletes and determine their associations with body composition and MetS. We sought to determine whether dietary intake and dietary protein source (i.e., animal- and plant-based) and quality were associated with MetS, as estimated by the Simple Method for Quantifying Metabolic Syndrome (siMS) score and the siMS risk score, and whether protein pacing was associated with body composition in Division III female athletes.

## 2. Materials and Methods

### 2.1. Participants

Division III female athletes between the ages of 18 and 25 years from Skidmore College were recruited via email and word of mouth. This cross-sectional study was conducted at Skidmore College in Saratoga Springs, NY, USA from August 2022 to December 2023. After providing written informed consent and completing a health history questionnaire, documents were screened by the research team and the director of Health Services at Skidmore College to determine whether participants were healthy and capable of partaking in the study. Participants were included if they were assigned female at birth and a Division III athlete at Skidmore College. Participants were excluded if they were pregnant, users of tobacco, e-cigarettes, or smokeless tobacco, had taken or were currently taking medications that could influence muscle size and strength (e.g., testosterone and growth hormone), were not vaccinated or boosted against COVID-19, had any orthopedic, musculoskeletal, or other health problem currently affecting their ability to practice or perform exercise, or had or were being treated for any neuromuscular, cardiovascular, or metabolic disease or cancer. Participants could also have been excluded depending on their previous radiation exposure in the 12 months prior to their participation in the study. The study was approved by the Skidmore College Institutional Review Board (#2203-1021).

### 2.2. Procedures

Each participant completed two individual sessions. Session 1 (S1) was completed via video conference (Zoom Video Communications, San Jose, CA, USA) to go over three-day dietary log training. Session two (S2) was conducted approximately seven days after S1, depending upon the availability of the X-ray technician, and was scheduled within one hour of waking, after a 12-h fast between 0600 and 0900. At S2, researchers collected completed three-day dietary logs and then assessed anthropometrics (i.e., height, body mass, and waist and hip circumference), resting blood pressure, metabolic and physiological variables using finger stick blood testing, and body composition using dual-energy x-ray absorptiometry (DXA). A certified X-ray technician conducted all DXA scans, while all other assessments were administered by trained research staff.

### 2.3. Dietary Intake Assessment

Dietary intake was measured via three-day dietary logs [37], analyzed using Food Processor version 11.6.522 (ESHA Research, Salem, OR, USA). Each participant underwent a 10 min dietary log training, completed by the lead researcher, that included food portioning handouts and serving size guides. Intake was collected on any two typical days and one atypical day (e.g., one weekend day) selected by the participant during a seven-day collection period.

### 2.4. Anthropometric and Hemodynamic Assessments

Height was measured using a stadiometer (Seca 213, Chino, CA, USA), and body mass was recorded using a digital scale (Befour, Saukville, WI, USA). Hip and waist circumferences were measured at the widest portion of the hips and at the umbilicus, respectively, using a spring-loaded Gulick measurement tape (Fabrication Enterprises, White Plains, NY, USA). Resting blood pressure was measured manually immediately following the DXA body composition scan in the supine position at the brachial artery using a sphygmomanometer (American Diagnostic Corporation, Hauppauge, NY, USA) and a stethoscope (Classic II SE; Littmann, 3M, Maplewood, MN, USA).

### 2.5. Blood Lipid Profile and Glucose

Blood samples were collected within one hour of waking between 0600 and 0900 in the Vascular Physiology and Metabolism Laboratory on the Skidmore College campus. Capillary blood from the fingertip was collected in a 40-microliter capillary tube and analyzed in a single-use cassette using the Cholestech LDX System (Abbott, San Diego, CA, USA), which has been documented to be valid and reliable [38,39,40]. Several health-related biomarkers (i.e., total cholesterol, high-density lipoprotein cholesterol, low-density lipoprotein cholesterol, triglycerides, and glucose) for each participant were analyzed. Quality controls, such as optics checks, and analyte controls were conducted on a regular basis and were all within normal limits. The coefficients of variation for level 1 and level 2 analyte controls were 6.6% and 5.6% for total cholesterol (TC), 13.4% and 10.6% for high-density lipoprotein (HDL) cholesterol, 6.5% and 4.8% for low-density lipoprotein (LDL) cholesterol, 2.7% and 2.3% for triglycerides, and 2.4% and 2.6% for glucose, respectively.

### 2.6. Body Composition

Total body mass, fat mass, lean mass, visceral adipose tissue, and body fat percentage were assessed using a GE Lunar iDXA (GE Medical, Madison, WC, USA). Participants were asked to dress in shorts and a T-shirt. For this procedure, two Velcro straps were placed on the participant’s lower limbs to help keep the lower body in the correct position (i.e., lying supine) during the scan. The scan took approximately 5–10 min depending on the height and body mass of the participant. When the scan was complete the Velcro straps were removed, and blood pressure was taken before the participant was assisted off the DXA table.

### 2.7. siMS Score and siMS Risk Score

Each of the five MetS risk factors (i.e., waist circumference, triglycerides, HDL, systolic blood pressure, and glucose) was calculated into the siMS score and the siMS risk score, which is calculated using the siMS score, age, and the family history risk factor definition of the International Diabetes Federation and the American Heart Association [5,41]. The siMS score and siMS risk score have been shown to be an accurate and simplified technique for evaluating and assessing MetS over time, even among younger adults not presenting with the condition [7,41,42].

### 2.8. Statistical Analysis

An a priori power analysis was performed with G*Power software version 3.1.9.7 (University of Düsseldorf, Düsseldorf, North Rhine-Westphalia, Germany). Using an effect size (ES) of 0.5, an α of 0.05, and a power of 0.8, it was estimated that 21 participants were needed to detect an association between dietary protein source and MetS, as assessed by siMS score and siMS risk score.

Energy and macronutrient intake values were compared to the updated recommendations of the International Society of Sports Nutrition (ISSN) for exercise and sports nutrition [43], and micronutrient intake was compared to the nutrient recommendations issued by the National Academies of Sciences, Engineering, and Medicine [44]. Food group targets were compared to the United States Department of Agriculture (USDA) MyPlate recommendations for a 2000-kilocalorie level [45]. To examine protein pacing, daily protein intake was divided into three mealtime periods: morning (before 11:30), afternoon (11:30–16:00), and evening (after 16:00). Protein pacing was defined as relative (≥0.4 g/kg) and absolute (≥30 g). Meeting protein pacing cut points during one mealtime period (i.e., either morning, afternoon, or evening) was recorded as “1”, and these were summed to create two ordinal variables each with four levels, achieving relative or absolute protein pacing at zero, one, two, or three mealtime periods.

Stepwise linear regression models were used to evaluate our research questions. First, we asked whether dietary intake and dietary protein source (i.e., animal- and plant-based) were associated with MetS, as estimated by the siMS score and the siMS risk score, in Division III female athletes. Age (yr), body mass (kg), body mass index (kg/m^2^), ratio of animal-based protein intake (ABP) to plant-based protein intake (PBP), ABP (g/d), PBP (g/d), ratio of high-quality to low-quality ABP (ABP QR), and relative energy (kcal/kg/d), protein (g/kg/d), carbohydrate (g/kg/d), and fat intake (g/kg/d) were included in these stepwise analyses. Second, whether protein pacing was associated with body fat percentage in Division III female athletes was assessed. Age, body mass, height (cm), ABP, PBP, ABP:PBP, ABP QR, relative energy, protein, carbohydrate, and fat intake, and relative (g/kg/meal) and absolute protein pacing (g/meal) were included in the regression. Lastly, independent sample two-tailed t-tests were used to assess differences between off-season and in-season Division III female athletes regarding their basic characteristics, nutritional intake, blood glucose and lipid profiles, body composition, and siMS score and siMS risk score. Levene’s test was used for equality of variance. If violations of the assumption of equal variances were found, a Welch test was used. These values were reported instead and indicated with an “^a^”. Effect sizes (ESs) are listed as Cohen’s *d*. All statistical analyses were performed using JASP version 0.19 (University of Amsterdam, Amsterdam, The Netherlands). All measures are reported as mean ± standard deviation (SD), and statistical significance was set at α = 0.05.

## 3. Results

### 3.1. Participant Characteristics

A total of 25 Division III female athletes (13 off-season; 12 in-season) between the ages of 18 and 22 years old were recruited for and completed this study. No differences were found between off-season (O) and in-season (I) athletes regarding their basic descriptive characteristics (Table 1). Two athletes (2 O) met the MetS risk factor criteria for waist circumference (i.e., >88 cm), and one athlete (1 I) met the criteria for systolic blood pressure (i.e., ≥130 mm Hg). The various sports and distribution of athletes per sport were basketball (1 O; 1 I), field hockey (1 I), lacrosse (2 O, 4 I), rowing (4 I), soccer (3 O), softball (3 O, 1 I), swimming (2 O), tennis (1 I), and volleyball (2 O).

### 3.2. Nutritional Intake Analysis

#### 3.2.1. Energy, Fiber, and Macronutrient Intake

There was a significant difference between O and I athletes for relative PBP intake (t_23_ = −3.166, *p* = 0.004; *d* = −1.267). The energy, fiber, and macronutrient intakes of the Division III female athletes are presented in Table 2.

On average, the athletes, both O and I, had an energy intake within the absolute intake range (i.e., 2000–7000 kcal/d) suggested by the ISSN [43]. However, once adjusted for body mass, the athletes failed to meet the relative intake range (i.e., 40–70 kcal/kg/d). The athletes had a carbohydrate intake within the absolute intake range (i.e., 250–1200 g/d) for moderate activity (i.e., 2–3 h per day of intense exercise performed 5–6 times per week) [43]. However, again, none of the athletes met the relative intake range (i.e., 5–8 g/kg/d) for moderate activity. The athlete’s percentage of daily energy intake from carbohydrates was 47% ± 8% (48% ± 8% O, 45% ± 8% I), which is within the Acceptable Macronutrient Distribution Range (AMDR) (i.e., 45–65% of kcal/d), but failed to meet the Adequate Intake (AI) for dietary fiber (i.e., 25 g/d). The athletes’ percentage of daily energy intake from lipids, aside from the O athletes, was 36% ± 7% (34% ± 7% O, 39% ± 6% I), which is greater than the AMDR (i.e., 20–35% of kcal/d). The athletes’ percentage of daily energy intake from saturated fat was 11% ± 4% (10% ± 3% O, 13% ± 4% I), which is greater than the < 10% recommended by the American Heart Association [46]. All 25 athletes (13 O, 12 I) met the minimum relative protein intake recommendation (i.e., 1.4 g/kg) according to the ISSN [43]. The athletes’ percentage of daily energy intake for proteins was 18% ± 4% (19% ± 5% O, 17% ± 4% I), which is within the AMDR (i.e., 10–35% kcal/d).

Individually, 16 athletes (7 O, 9 I) had an energy intake within the absolute intake range, while only five athletes (3 O, 2 I) were within the relative intake range. Only 11 athletes (7 O, 4 I) had a carbohydrate intake within the absolute intake range for moderate activity, while only five athletes (2 O, 3 I) were within the relative intake range for moderate activity (i.e., 5–8 g/kg/d), and one athlete (1 O) was within the relative intake range for high activity (i.e., 8–10 g/kg/d). Eleven athletes (6 O, 5 I) met the AI for dietary fiber. Twelve athletes (9 O, 3 I) were within the AMDR for lipid intake, while the remaining athletes were above. A total of 16 athletes (7 O, 9 I) had a saturated fat intake greater than 10% of their total energy intake. Last, 18 athletes (11 O, 7 I) met the minimum relative protein intake recommendation (i.e., 1.4 g/kg).

#### 3.2.2. Micronutrient Intake

A significant difference between O and I athletes was observed for vitamin E (t_23_ = −3.924, *p* < 0.001, *d* = −1.571). The micronutrient intake of the Division III athletes, including the Dietary Reference Intakes (DRIs) of these micronutrients, can be found in Table 3. The athletes failed to meet the Recommended Dietary Allowance (RDA) for calcium (~90% of DRI), iron (~94%), magnesium (~89%), vitamin D (~33%), and vitamin E (~33%) and the AI for potassium (~98%) on average. The athletes did, however, meet the RDA for vitamin A (~135%), vitamin B_12_ (~154%), and vitamin C (~133%) and the AI for vitamin K (~230%) and sodium (~241%). The athletes’ sodium intake was greater than the 2300 mg/d recommended for chronic disease risk reduction [44]. Of greater concern were the athletes individually, as indicated by the large standard deviations listed in Table 3. Individually, 10 athletes (4 O, 6 I) met the RDA for calcium (304.1 mg min, 1669.0 mg max), 9 (5 O, 4 I) for iron (7.2 mg min, 34.7 mg max), 8 (3 O, 5 I) for magnesium (169.9 mg min, 431.5 mg max), 14 (7 O, 7 I) for vitamin A (121.8 mcg min, 2755.6 mcg max), 15 (6 O, 9 I) for vitamin B_12_ (0.4 mcg min, 10 mcg max), 14 (7 O, 7 I) for vitamin C (14.6 mg min, 203.3 mg max), 1 (1 I) for vitamin D (0.1 mcg min, 53.9 mcg max), and 1 (1 I) for vitamin E (1.7 mg min, 15.5 mg max), while 11 athletes (6 O, 5 I) met the AI for potassium (1237.3 mg min, 4045.4 mg max), 25 (13 O, 12 I) for sodium (1520.4 mg min, 7605.4 mg max), and 18 (10 O, 8 I) for vitamin K (18.5 mcg min, 650.1 mcg max). Of concern, 22 athletes (11 O, 11 I) consumed greater than 2300 mg/d of sodium.

#### 3.2.3. USDA MyPlate Recommendations

**Figure 1:**
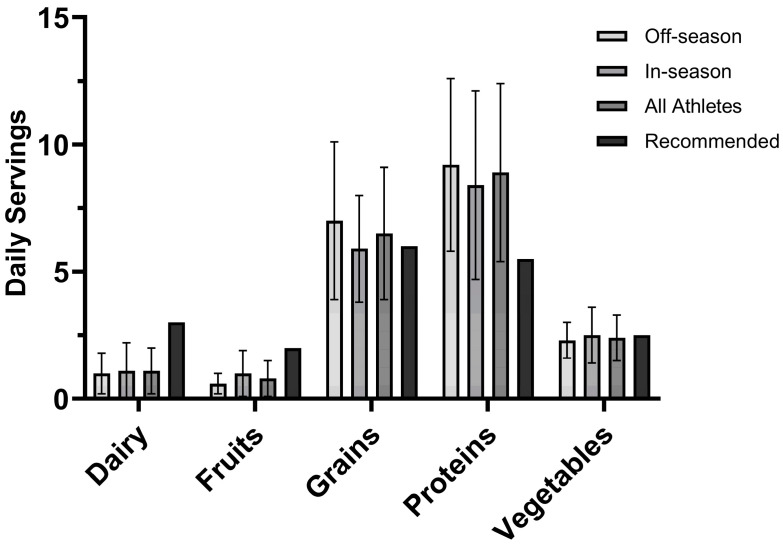
Daily food group servings of Division III female athletes (n = 25) compared to USDA recommended daily amounts for a 2000-kilocalorie level.

The athletes failed to meet the daily intake values for dairy (i.e., 3 cups), fruits (i.e., 2 cups), and vegetables (i.e., 2.5 cups), but met intake values for grains (i.e., 6 oz), except in-season athletes, and proteins (i.e., 5.5 oz). No statistical differences were observed between the athletes’ daily servings for dairy (t_23_ = −0.057, *p* = 0.955, *d* = −0.023), fruit (t_15_ = −1.376, *p* = 0.189, *d* = −0.558) ^a^, grains (t_23_ = 1.071, *p* = 0.295, *d* = 0.429), proteins (t_23_ = 0.562, *p* = 0.579, *d* = 0.225), or vegetables (t_23_ = −0.505, *p* = 0.619, *d* = −0.202). These values can be seen in Figure 1. Individually, the number of athletes that met the daily intake value for dairy was 1 (1 I), for fruits was 2 (2 I), for grains was 12 (8 O, 4 I), for proteins was 21 (11 O, 10 I), and for vegetables was 9 (4 O, 5 I).

#### 3.2.4. Protein Pacing

**Figure 2:**
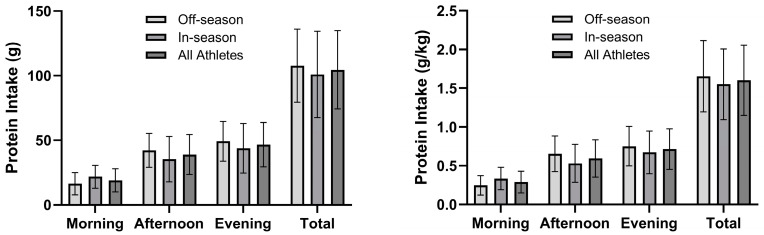
Daily absolute (g) and relative (g/kg) protein intakes per mealtime period (i.e., morning, afternoon, and evening) of Division III female athletes (n = 25).

No statistical difference was observed between athletes for absolute protein pacing (t_20_ = −0.396, *p* = 0.696, *d* = −0.160) ^a^ or relative protein pacing (t_23_ = 0.249, *p* = 0805, *d* = −0.100). The athletes met 1.8 ± 0.9 (1.8 ± 0.7 O, 1.9 ± 1.1 I) mealtime periods for absolute protein pacing (i.e., 30 g/meal) and 1.9 ± 0.8 (1.9 ± 0.8 O, 1.8 ± 1.0 I) mealtime periods for relative protein pacing (i.e., 0.4 g/kg/meal) (Figure 2). Two athletes (1 O, 1 I) did not meet absolute protein pacing (i.e., 30 g/meal) for any mealtime period, whereas 6 athletes (2 O, 4 I) met one mealtime period, 11 athletes (9 O, 2 I) met two mealtime periods, and 6 athletes (1 O, 5 I) met all three mealtime periods. Regarding relative protein pacing, 2 athletes (1 O, 1 I) did not meet any mealtime period, 5 athletes met one mealtime period (1 O, 4 I), 12 athletes (9 O, 3 I) met two mealtime periods, and 6 athletes (2 O, 4 I) met all three mealtime periods. Importantly, 20 athletes (12 O, 8 I) for absolute protein pacing and 18 athletes (11 O, 7 I) for relative protein pacing failed to achieve intakes for the morning mealtime period.

### 3.3. Body Composition

According to reference standards for body fat percentage using DXA, all athletes and off-season athletes fell within the 60th to 70th percentile, whereas the in-season athletes fell within the 50th to 60th percentile [47]. Individually, four athletes (3 O, 1 I) fell within the 90th to 100th, one (1 O) within the 80th to 90th percentile, four (2 O, 2 I) within the 70th to 80th percentile, three (2 O, 1 I) within the 60th to 70th, seven (2 O, 5 I) within the 50th to 60th, three (1 O, 2 I) within the 40th to 50th, two (1 O, 1 I) within the 30th to 40th percentile, and one (1 O) within the 20th to 30th percentile [47]. It is important to note that 13 athletes (6 O, 7 I) were below the age range, 20 to 29 years, for which these reference standards were taken. Additionally, the percentiles utilized were established from a primarily Caucasian cohort, which may not accurately represent all athletes in this study due to ethnic variation in body composition. Body composition data can be found in Table 4.

### 3.4. Blood Glucose and Lipid Profiles

Twenty-four athletes (13 O, 11 I) were included in the blood glucose, lipid profile, and siMS score and siMS risk score analyses, as one in-season athlete experienced nausea and light-headedness during the capillary blood draw, via finger prick, which prevented us from obtaining a sample. A significant difference between O and I athletes was observed for triglycerides (t_13_ = −3.573, *p* < 0.003, *d* = −1.505) ^a^. One athlete (I O) met the MetS risk factor criterion for triglycerides (i.e., ≥150 mg/dL). Five athletes (4 O, 1 I) met the MetS risk factor criterion for HDL cholesterol (i.e., <50 mg/dL) while ten athletes (7 O, 3 I) had HDL values above 60 mg/dL, which would be considered a negative CVD risk factor. These values are listed in Table 5.

### 3.5. siMS Score and siMS Risk Score

A significant difference between O and I athletes was observed for siMS Score (t_22_ = −2.237, *p* = 0.036, *d* = −0.917). The siMS score and siMS risk score values are listed in Table 6.

### 3.6. Regression Analyses

#### 3.6.1. MetS and Dietary Protein Source and Quality

**Figure 3:**
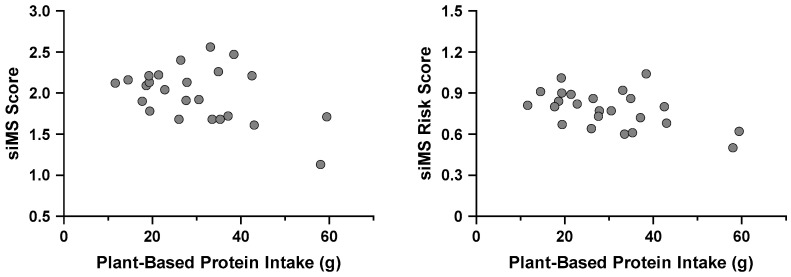
Greater plant-based protein intake is associated with lower siMS scores and siMS risk scores in Division III female athletes (n = 24). Abbreviation: siMS, Simple Method for Quantifying Metabolic Syndrome.

Only PBP was predictive for the siMS score and siMS risk score. There was an inverse relationship between PBP intake in both siMS score and siMS risk score (Figure 3). For every 1 g increase in PBP, the siMS score was lowered by 0.012, and the siMS risk score was lowered by 0.006. The regression model for the siMS score explained 20% of the variance in siMS scores (F_1, 22_ = 5.498, R^2^ = 0.200, adjusted R^2^ = 0.164, *p* = 0.028). The regression model for the siMS risk score explained 26% of the variance in siMS risk scores (F_1, 22_ = 7.614, R^2^ = 0.257, adjusted R^2^ = 0.223, *p* = 0.011).

#### 3.6.2. Body Composition, Dietary Protein Source and Quality, and Protein Pacing

**Figure 4:**
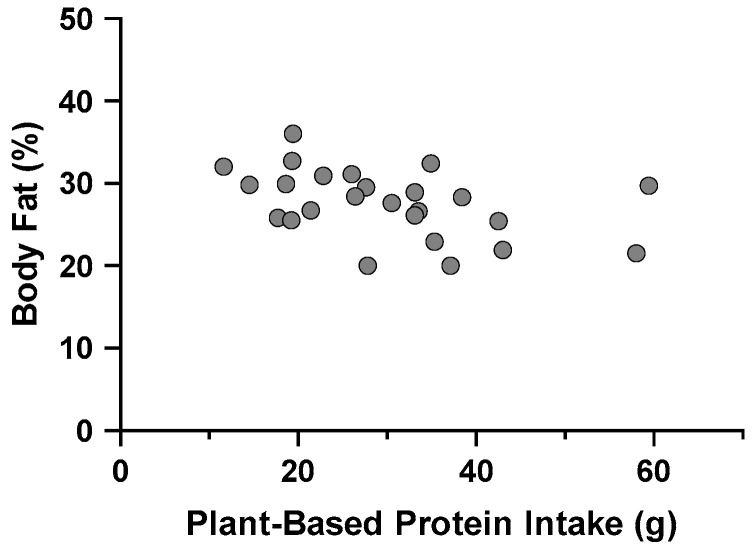
Plant-based protein intake is associated with reduced body fat percentage in Division III female athletes (n = 25).

Only PBP was predictive of body fat percentage. There was an inverse relationship between PBP and body fat percentage (Figure 4). For every 1 g of PBP, the body fat percentage was reduced by 0.148%. The regression model explained 19% of the variance in body fat percentages (F_1,23_ = 5.429, R^2^ = 0.191, adjusted R^2^ = 0.156, *p* = 0.029). The full breakdown of the stepwise regression determinants for siMS score, siMS risk score, and body fat percentage is found in Table 7.

## 4. Discussion

The purpose of this investigation was to assess the current dietary habits of Division III female athletes and determine their associations with body composition and MetS. Secondly, we sought to determine whether dietary intake and dietary protein source (i.e., animal- and plant-based) and quality were associated with MetS, as estimated by the siMS score and the siMS risk score, and whether protein pacing was associated with body composition in Division III female athletes. The primary finding of this study was that PBP intake had a significant inverse relationship with the siMS score and siMS risk score, while ABP, regardless of quality, was unrelated. Secondly, PBP intake had a significant inverse relationship with body fat percentage.

### 4.1. MetS Risk of Division III Female Athletes

Despite athletic status, some athletes displayed positive risk factors for MetS (i.e., large waist circumference, high systolic blood pressure and triglycerides, and attenuated HDL cholesterol). In fact, instances of positive MetS risk factors were 100% greater in the off-season athletes compared to the in-season athletes (6 O, 3 I). Interestingly, however, in-season athletes had significantly higher siMS scores, likely due to their significantly higher triglyceride levels (69.1%). We believe this may be a consequence of the quality of their ABP sources, which contributed to slightly higher lipid and saturated fat intake. For instance, in-season athletes consumed 51.2% of their ABP from red and processed meats and other fatty sources (e.g., fried chicken, cream cheese, and ice cream), compared to 38.3% for off-season athletes. Increased consumption of plant-based foods has long been associated with many cardiometabolic benefits, including lower risk of MetS, when implemented properly [20,48,49]. These health benefits are attributed to the increased nutritional quality of plant-based foods and simultaneous removal of animal-based products, specifically red and processed meats, as these have been associated with a greater risk of developing type II diabetes, CVD, and certain cancers [20,48]. However, the lack of nutritional education and access to sports dieticians for college athletes leads to concerns about the potential negative effects of replacing high-quality ABP, such as light skinless chicken, fish, egg whites, very lean cuts of beef, and skim milk, with PBP sources, as these resources are key for successful dietary adjustments [28].

PBP sources are less bioavailable for micronutrients such as iron, calcium, and vitamin B_12_ compared to ABP, which becomes a major concern for female athletes at risk of deficiency due in part to their typical diet, menstrual cycles, and potential low energy consumption [16,19,50]. Moreover, athletes require 1.3 to 1.7 times more dietary iron compared to non-athletes [50]. This poses a significant challenge, because the iron in plant-based foods is not as easily absorbed as the heme iron in animal products. Additionally, certain compounds in plants, such as polyphenols, tannins, and phytates, can further hinder iron absorption [50]. Microorganisms such as bacteria and fungi synthesize vitamin B_12_ in the gastrointestinal tract of animals, making them the only natural source of this essential nutrient [50]. Consequently, vitamin B_12_ is predominantly obtained from animal products like meat, milk, and eggs in human diets. Since plants do not provide a reliable or adequate source of vitamin B_12_, athletes following plant-based diets often rely on fortified foods but ultimately need to supplement to meet their daily requirements. Collards, almonds, navy beans, and edamame can serve as good calcium sources, but the oxalate and phytate content of plant-based foods may hinder absorption, making it challenging to consume enough [50]. For more specific information, interested readers should consult an excellent review by Heller [50], which outlines the micronutrient needs and concerns of plant-based athletes, as well as a review by Rogerson [51], which covers nutritional recommendations for athletes and exercisers following vegan diets.

Thus, this study’s finding that ABP intake, regardless of quality, was not associated with siMS score or siMS risk score is incredibly important, as it suggests female athletes may receive cardiometabolic benefits by simply consuming more PBP without needing to remove ABP sources from their diet and consequently contributing to nutrient deficiency. However, it is also important to acknowledge that low-quality ABPs are associated with a greater risk of cardiometabolic disease. Therefore, longitudinal studies assessing the quality of ABP intake in athletes may reveal their influence on siMS scores and siMS risk scores over time. While there are no normative values regarding the siMS score, it has been associated with long-term risk of coronary heart disease, myocardial infarction, and CVD and all-cause mortality and may be used as a means of estimating the degree of cardiometabolic burden in individuals without overt MetS [42]. These data highlight that dietary habits (e.g., lower intake of PBP sources), even in athletes, may place them at greater risk for MetS, which is critical, as the habits of young adults often translate into their lifespan. Further monitoring may be indicated for Division III athletes to ensure adequate nutritional status for sport but also guidance for transition into a healthy post-athletic life.

### 4.2. Dietary Intake of Division III Female Athletes

One point to address is the athletes’ percentage of daily energy intake from lipids, which was greater than the AMDR (i.e., 20–35% of kcal/d). While it is recommended that athletes consume a moderate amount of lipids (i.e., 30%), proportions up to 50% can be safely ingested by athletes during regular high-volume training [43]. A greater concern was the athletes’ percentage of daily energy intake from saturated fat. High saturated fat intake is shown to elevate LDL cholesterol and is associated with a 9–13% higher mortality [52]. This may be confounded by the athletes’ inability to meet the AI for dietary fiber, even though they achieved more than the recommended daily servings of grains for a 2000-kilocalorie diet. Fiber has long been associated with lower rates of CVD and type 2 diabetes [46,53], and soluble fiber specifically plays a key role in the bile reuptake loop, reducing the reabsorption of bile acid in the gut, ultimately increasing the synthesis of bile acids from cholesterol and thus reducing cholesterol in circulation [54]. Thus, athletes may benefit by ensuring at least half of their grains come from whole grains (e.g., quinoa, brown rice).

In the present study, athletes failed to meet the RDA for calcium, iron, magnesium, vitamin D, and vitamin E and the AI for potassium, which may likely be attributed to an overall low energy and carbohydrate intake stemming from inadequate intakes of dairy, fruits, and vegetables. This may pose a serious risk to the health and performance of these athletes, especially if these are not compensated for during the transition to in-season, and specifically because the athletes in the present study likely had greater energy and macro- and micronutrient needs than those used for assessment [53,55,56]. Low carbohydrate intake has been shown to have negative effects on bone, immunity, and iron biomarkers, even in the absence of low energy availability [55], and can contribute to loss of muscle glycogen, lean body mass, and strength [53,55]. Low intake of iron can reduce tissue oxidative capacity and oxygen-carrying capacity, impairing the overall aerobic capacity of these athletes [50,53]. Vitamin E, along with vitamin C, possess antioxidant properties that may help athletes endure increased training by mitigating oxidative damage [43]. Calcium, of course, is important for bones, but also nerve and muscle function, intracellular signaling, and hormonal secretion [50]. Combined with vitamin D and magnesium, the three are incredibly important for bone health and optimal nerve and muscle function [50,57]. Magnesium also plays an important role in regulating blood pressure and has been studied as an ergogenic aid due to its involvement in cellular energy production and storage, particularly in maintaining blood glucose levels [57]. Deficient potassium intake likely contributes to muscle and whole-body fatigue, impairing exercise performance. However, chronic and acute changes in arterial plasma potassium levels, such as hypokalemia, can have serious health implications for cardiac function [58].

Unfortunately, it is quite common for athletes to fail at meeting the higher energy and macro- and micronutrient requirements needed to support their increased physical activity and exercise [16,18,19,28], which also causes concern regarding the availability of high-quality amino acids essential for the repair and support of muscle mass and strength. Low energy intake has long been associated with loss of muscle mass [59,60,61], and recent literature has shown low energy availability in female athletes to reduce myofibrillar and sarcoplasmic muscle protein synthesis [60]. While this reduction may be attenuated acutely with resistance training, adequate protein ingestion becomes increasingly important to combat the negative consequences this may have on skeletal muscle adaptation [53,59,60,62,63].

### 4.3. Body Composition, Dietary Protein Source and Quality, and Protein Pacing of Division III Female Athletes

In this study, athletes had no trouble achieving relative protein intakes (i.e., 1.65 g/d) sufficient for building and maintaining muscle mass, even though overall energy intake was apparently low [43]. However, most athletes in this study failed to meet adequate energy and protein intake during morning hours (i.e., before 11:30) and across all three mealtime periods. Thus, there may be potential to maximize muscle protein synthesis, although optimal protein doses for athletes vary and are influenced by age and recent resistance exercise [43]. Additionally, proteins differ based on their source, amino acid profile, and the methods of processing or isolating, affecting whole-body catabolism, anabolism, and acute muscle protein synthesis [43]. So, while not essential for increased performance and muscular adaptations, it is generally recommended to consume protein every 3–4 h across four to six meals daily, mainly from high-quality ABP sources [34,35,36,43]. Though, some studies have shown pacing proteins across just three mealtime periods (i.e., morning, afternoon, and evening) to be associated with greater lean mass and muscle strength in healthy females, which could lead to improved cardiometabolic outcomes and impact sport-specific performance [35,36,43,64].

Interestingly, we observed that increased PBP intake, and not protein pacing, contributed to a reduced body fat percentage in this study. While protein intake primarily from plant-based sources can adequately support muscle and bone health [20,48,49], athletes should avoid indiscriminately replacing ABP sources, as this may lead to increased frequency of injury and muscle wasting due to inadequate protein intake [20,22,43,65]. For example, athletes tend to meet general dietary protein recommendations (i.e., 0.8 g/kg) while on plant-based diets but struggle to meet the minimum protein requirements (1.2–2.0 g/kg) shown to improve and support muscle hypertrophy and strength [66]. This is of concern because greater amounts of PBP (i.e., beyond the minimum requirements) may be needed to maximize muscle protein synthesis due to the digestible indispensable amino acid score differentiation compared to that of ABP, especially leucine [7,62,65,66]. Again, this further emphasizes the importance that ABP intake was not associated with the siMS score or siMS risk score and body fat percentage, as it suggests female athletes may not need to immediately replace low-quality ABP sources in their diet. Though, we would encourage these athletes to ensure most of their ABP comes from high-quality sources like light skinless chicken, fish, egg whites, lean cuts of beef, and skim milk.

### 4.4. Limitations

This study has several limitations to address. First, our study’s findings may not be generalizable to other divisions due to potential differences in dietary support and resources and a relatively modest sample size. Additionally, since we recruited athletes from various sports, individual nutritional needs and subsequent risks may vary. A strength of the study is that our sample represented 13.4% of the Division III female athletes at Skidmore College. Second, the use of self-reported dietary intake logs may have led to underreporting of total energy and dietary intake, as evidenced by major US recovery biomarker studies showing underreporting by 6–16% for energy intake and lower underreporting for some nutrients (e.g., protein 5% and potassium 3%) [67,68,69,70]. More objective measures, such as biomarker validation or controlled feeding, could have enhanced the accuracy of our nutrition data and may have indicated that the athletes met minimum relative energy and carbohydrate recommendations and some micronutrient DRIs. However, these athletes still consumed excess lipids and saturated fat and likely had greater energy and nutrient needs than assessed. Biomarker validation was not possible for our study due to availability, expense, and logistical challenges, and controlled feeding studies have shown minimal differences between self-reported and known intakes, suggesting low levels of underreporting [68,71]. Thus, we utilized self-reported dietary intake logs for their accessibility and to minimize participant burden, considering practical limitations like busy schedules and travel. Despite these limitations, self-reported intakes tend to be less biased than food frequency questionnaires, offer more detail on dietary habits (i.e., a major focus of this study), and are culturally inclusive [67,68]. An additional strength of this process is that most of our athletes relied on Skidmore College Dining Services for their dietary intake. Detailed recipes for meal items were provided to us by the executive and assistant chef of dining services, which enhanced the overall quality of our analysis. Third, some athletes’ metabolic values for TC (n = 2) and triglycerides (n = 5) were lower than the minimal acceptable values (i.e., 100 and 45 mg/dL, respectively) measurable by the Cholestech LDX analyzer. Thus, to ensure data consistency and avoid bias from excluding these data points, we decided to use the minimal acceptable values instead. This approach helped us maintain the sample size, preserving the study’s power and validity. Additionally, it may allow for standardization, facilitating comparison with future studies.

### 4.5. Future Research Directions

Future studies should measure physical activity and exercise energy expenditure to determine the risk of relative energy deficiency of these athletes to more accurately assess nutritional needs and their potential consequences on cardiometabolic health and MetS risk. Second, studies focusing exclusively on individual sports or metabolic types would undoubtedly enhance the practical applicability of these results and could lead to more personalized dietary recommendations for athletes, while also aiming to replicate these findings with larger and more diverse cohorts, potentially across multiple institutions. Third, longitudinal studies on the dietary intake of athletes, including the source and quality of ABP and PBP, may reveal causal relationships between dietary habits and MetS risk or onset. This is important, as low-quality ABP, such as red and processed meats, has been associated with a greater risk of CVD. Fourth, studies should implement and evaluate targeted nutritional education programs, as they may provide practical solutions that will help these athletes improve their dietary habits and reduce MetS risk over the athlete’s lifespan. Alternatively, the National Collegiate Athletic Association (NCAA) could increase its outreach to athletes across all divisions (I-III) to improve nutritional education from both health and performance perspectives. Although a recent narrative review revealed that collegiate athletes have poor knowledge of nutrition, and thus a need for sports dieticians or better outreach by the NCAA, there has been little work on the intervention side [13]. Researchers and organizations could consider the NCAA coordinating with registered dieticians, dining services on campus, coaches, and academics (e.g., credit-bearing coursework emphasizing health and nutrition) to positively impact the nutritional knowledge and practices of athletes. Lastly, our findings can serve as the basis for future research to explore the underlying causes of these nutrient deficiencies and dietary trends, as well as potential interventions, while employing more objective measures of dietary intake, such as biomarker validation, to better assess the accuracy of dietary data.

## 5. Conclusions

In conclusion, our findings show that Division III female athletes tend to have a lower energy intake and consume fewer fruits, vegetables, and dairy, subsequently leading to lower intakes of macro- and micronutrients. Additionally, athletes may experience a reduced body fat percentage, and its associated health benefits, by increasing their consumption of PBP. The increase in PBP may help these athletes reduce MetS risk without needing to remove high-quality ABP sources from their diet, making it easier for them to meet the increased nutritional requirements to support their physiological demands without increasing the risk of or exacerbating nutrient deficiency. Of course, metabolic differences, physical activity levels, and practical limitations among athletes necessitate individualized nutrient requirements and timing adjustments. This complexity underscores the need for all athletes to have access to a sports dietitian.

## Figures and Tables

**Table 1:** Descriptive characteristics of Division III female athletes.

Variables	Total (n = 25)	Off-Season (n = 13)	In-Season (n = 12)	*p* Value	ES *d*
Age (years)	19.6 ± 1.3	19.7 ± 1.4	19.4 ± 1.2	0.605	0.210
Height (cm)	167.5 ± 6.9	169.3 ± 5.5	165.5 ± 7.9	0.165	0.575
Body mass (kg)	65.9 ± 7.0	66.4 ± 8.4	65.3 ± 5.3	0.714	0.149
BMI (kg/m^2^)	23.5 ± 2.0	23.1 ± 2.1	23.9 ± 1.8	0.309	−0.416
Hip C (cm)	95.8 ± 9.1	94.5 ± 12.0	97.3 ± 4.2	0.427 ^a^	−0.322
Waist C (cm)	76.8 ± 6.6	78.3 ± 8.5	75.1 ± 3.4	0.222 ^a^	0.501
Waist-to-hip ratio	0.8 ± 0.2	0.9 ± 0.2	0.8 ± 0.0	0.218 ^a^	0.509
SBP (mmHg)	112.8 ± 9.6	112.8 ± 8.9	112.9 ± 10.8	0.970	−0.015
DBP (mmHg)	69.1 ± 6.8	67.3 ± 6.2	71.0 ± 7.2	0.182	−0.551

Data are presented as mean ± standard deviation. Abbreviations: BMI, body mass index; C, circumference; SBP, systolic blood pressure; DBP, diastolic blood pressure. ^a^ Levene’s test was significant (*p* < 0.050), Welch test was used.

**Table 2:** Energy, fiber, and macronutrient intake of Division III female athletes.

Variables		Total (n = 25)	Off-Season (n = 13)	In-Season (n = 12)	*p* Value	ES *d*
Energy (kcal)	A (/d)	2280.1 ± 568.3	2249.9 ± 597.8	2312.8 ± 559.0	0.789	−0.109
	R (/kg/d)	35.0 ± 9.4	34.6 ± 11.1	35.4 ± 7.7	0.838	−0.083
Carbs (g)	A (/d)	267.1 ± 84.1	271.3 ± 89.9	262.5 ± 81.1	0.799	0.103
	R (/kg/d)	4.1 ± 1.4	4.2 ± 1.6	4.0 ± 1.1	0.778	0.114
Dietary fiber (g)	A (/d)	23.9 ± 8.9	23.5 ± 6.6	24.3 ± 11.2	0.842	−0.081
	R (/kg/d)	-	-	-	-	-
Lipids (g)	A (/d)	91.9 ± 27.1	85.7 ± 28.0	98.6 ± 25.6	0.242	−0.481
	R (/kg/d)	1.4 ± 0.4	1.3 ± 0.5	1.5 ± 0.4	0.284	−0.439
Saturated fat (g)	A (/d)	28.7 ± 10.4	25.9 ± 10.0	31.7 ± 10.4	0.174	−0.561
	R (/kg/d)	-	-	-	-	-
Proteins (g)	A (/d)	104.5 ± 30.3	107.7 ± 28.2	101.0 ± 33.3	0.590	0.219
	R (/kg/d)	1.6 ± 0.5	1.7 ± 0.5	1.6 ± 0.5	0.577	0.226
ABP (g)	A (/d)	71.7 ± 28.2	75.9 ± 28.2	67.3 ± 28.7	0.456	0.304
	R (/kg/d)	1.5 ± 0.9	1.2 ± 0.4	1.9 ± 1.2	0.097 ^a^	−0.726
PBP (g)	A (/d)	30.0 ± 12.2	29.2 ± 12.2	30.9 ± 12.6	0.741	−0.134
	R (/kg/d)	0.6 ± 0.3	0.4 ± 0.2	0.8 ± 0.3	0.004 ^^^	−1.267
ABP:PBP	-	2.8 ± 1.6	3.1 ± 1.7	2.5 ± 1.5	0.379	0.359
ABP QR	-	1.9 ± 1.8	2.4 ± 2.0	1.4 ± 1.4	0.194	0.414
ABP HQ (%)	-	55.5 ± 19.9	61.7 ± 17.0	48.8 ± 21.4	0.107	−0.671

Data are presented as mean ± standard deviation. Abbreviations: Carbs, carbohydrates; A, absolute; R, relative; ABP, animal-based protein; PBP, plant-based protein; QR, quality ratio; HQ, high quality. ^^^ Indicates a significant difference between off-season and in-season athletes. ^a^ Levene’s test was significant (*p* < 0.050), Welch test was used.

**Table 3:** Micronutrient intake of Division III female athletes.

Variables	DRI	Total (n = 25)	Off-Season (n = 13)	In-Season (n = 12)	*p* Value	ES *d*
Calcium (mg)	1000 ^#^	905.1 ± 365.2	873.6 ± 357.0	939.2 ± 386.7	0.663	−0.177
Iron (mg)	18 ^#^	17.0 ± 6.6	16.9 ± 7.5	17.0 ± 5.7	0.989	−0.006
Magnesium (mg)	310 ^#^	280.1 ± 73.7	280.0 ± 66.4	280.2 ± 83.8	0.996	−0.002
Potassium (mg)	2600 *	2551.9 ± 790.1	2527.9 ± 720.2	2577.8 ± 891.5	0.879	−0.062
Sodium (mg)	1500 *	3622.7 ± 1352.1	3522.4 ± 1595.4	3731.2 ± 1089.6	0.708	−0.152
Vitamin A (mcg)	700 ^#^	946.3 ± 643.6	847.4 ± 451.7	1053.4 ± 810.6	0.436	−0.317
Vitamin B_12_ (mcg)	2.4 ^#^	3.7 ± 2.6	3.1 ± 2.5	4.4 ± 2.6	0.226	−0.499
Vitamin C (mg)	75 ^#^	100.0 ± 61.5	90.3 ± 48.3	110.6 ± 74.0	0.430 ^a^	−0.326
Vitamin D (mcg)	15 ^#^	5.0 ± 10.6	2.7 ± 3.3	7.5 ± 14.8	0.270	−0.452
Vitamin E (mg)	15 ^#^	5.1 ± 3.2	3.2 ± 1.6	7.2 ± 3.3	<0.001 ^^^	−1.571
Vitamin K (mcg)	90 *	207.3 ± 170.1	202.4 ± 149.8	212.5 ± 196.5	0.886	−0.058

Data are presented as mean ± standard deviation. Abbreviations: DRI, Dietary Reference Intake. ^#^ DRI expressed as Recommended Dietary Allowance. * DRI expressed as Adequate Intake. ^^^ Indicates a significant difference between off-season and in-season athletes. ^a^ Levene’s test was significant (*p* < 0.050), Welch test was used.

**Table 4:** Body compositions of Division III female athletes.

Variables	Total (n = 25)	Off-Season (n = 13)	In-Season (n = 12)	*p* Value	ES *d*
Tissue mass (kg)	63.3 ± 6.8	63.7 ± 8.1	62.8 ± 5.3	0.735	0.137
Fat mass (kg)	19.5 ± 10.4	21.0 ± 14.4	17.8 ± 2.0	0.434 ^a^	0.318
Lean mass (kg)	45.7 ± 4.5	46.3 ± 4.3	45.0 ± 4.8	0.499	0.275
Visceral fat mass (kg)	0.1 ± 0.1	0.1 ± 0.1	0.1 ± 0.1	0.860	−0.072
BMD (g∙cm^2^)	1.2 ± 0.1	1.2 ± 0.1	1.2 ± 0.1	0.847	0.078
Body fat tissue (%)	27.6 ± 4.1	26.9 ± 5.0	28.4 ± 3.0	0.378	−0.360

Data are presented as mean ± standard deviation. Abbreviations: BMD, bone mineral density. ^a^ Levene’s test was significant (*p* < 0.050), Welch test was used.

**Table 5:** Blood glucose and lipid profiles of Division III female athletes.

Variables	Total (n = 24)	Off-Season (n = 13)	In-Season (n = 11)	*p* Value	ES *d*
Glucose (mg/dL)	85.3 ± 6.4	86.7 ± 6.4	83.6 ± 6.2	0.249	0.485
Triglycerides (mg/dL)	78.0 ± 33.1	59.2 ± 15.3	100.1 ± 35.2	0.003 ^^,a^	−1.505
T cholesterol (mg/dL)	152.5 ± 28.9	147.8 ± 30.2	158.1 ± 27.7	0.399	−0.352
LDL (mg/dL)	79.6 ± 21.2	77.5 ± 20.6	82.1 ± 22.6	0.605	−0.215
HDL (mg/dL)	57.4 ± 13.1	58.6 ± 17.3	55.9 ± 5.9 ^a^	0.604 ^a^	0.210
Non-HDL (mg/dL)	95.2 ± 23.0	89.2 ± 20.7	102.2 ± 24.6	0.175	−0.574

Data are presented as mean ± standard deviation. Abbreviations: HDL, high-density lipoprotein; LDL, low-density lipoprotein; T, Total. ^^^ Indicates a significant difference from off-season athletes. ^a^ Levene’s test was significant (*p* < 0.050), Welch test was used.

**Table 6:** siMS Score and siMS Risk Score of Division III female athletes.

Variables	Total (n = 24)	Off-Season (n = 13)	In-Season (n = 11)	*p* Value	ES *d*
siMS score	1.99 ± 0.33	1.86 ± 0.29	2.14 ± 0.30	0.036 ^^^	−0.917
siMS risk score	0.78 ± 0.14	0.73 ± 0.12	0.84 ± 0.13	0.050	−0.850

Data are presented as mean ± standard deviation. Abbreviations: siMS, Simple Method for Quantifying Metabolic Syndrome. ^^^ Indicates a significant difference from off-season athletes.

**Table 7:** Stepwise regression determinants for siMS score, siMS risk score, and body fat percentage.

	β ± SE	*p*-Value	R^2^	Adjusted R^2^
**siMS score**			0.200	0.164
Constant	2.339 ± 0.161	<0.001		
PBP (g)	−0.012 ± 0.005	0.028		
**siMS risk score**			0.257	0.223
Constant	0.947 ± 0.064	<0.001		
PBP (g)	−0.006 ± 0.002	0.011		
**Body fat percentage**			0.191	0.156
Constant	32.031 ± 2.053	<0.001		
PBP (g)	−0.148 ± 0.064	0.029		

Abbreviations: β, beta; SE, standard error; PBP, plant-based protein; siMS, Simple Method for Quantifying Metabolic Syndrome.

## Data Availability

The data presented in this study are available on request from the corresponding authors as permissions from college IRB have not been obtained for data sharing.
